# Anorectal malignant melanoma: curative abdominoperineal resection: patient selection with 18F-FDG-PET/CT

**DOI:** 10.1186/s12957-016-0938-x

**Published:** 2016-07-15

**Authors:** Claudius Falch, Sven Mueller, Andreas Kirschniak, Manuel Braun, Alfred Koenigsrainer, Bernhard Klumpp

**Affiliations:** Department of Surgery and Transplantation, University of Tuebingen, Hoppe-Seyler-Strasse 3, 72076 Tuebingen, Germany; Department of Diagnostic and Interventional Radiology, University of Tuebingen, Tuebingen, Germany

**Keywords:** Anal melanoma, Rectal melanoma, Anus neoplasms, Colorectal surgery, Abdominoperineal resection, Computed tomography, PET

## Abstract

**Background:**

Anorectal malignant melanomas (ARMM) are rare tumors, characterized by an early lymphatic spread and distant metastasis, resulting in an extremely poor overall survival. The objective of this study was to determine the pattern of regional lymph node metastasis (LNM) by computed tomography (CT) and 18F-FDG-PET/CT in patients undergoing abdominoperineal resection (APR) and its impact on oncologic outcome.

**Methods:**

A retrospective analysis of six consecutive patients who underwent APR due to primary ARMM was performed. Patients were staged by CT and PET/CT.

**Results:**

Four out of six patients had preoperative LNM involvement (two patients inguinal and perirectal, one iliacal, one perirectal), with two of them presenting with distant metastases additionally. Inguinal/iliacal LNM in two patients as well as liver metastasis in one patient was seen in PET/CT and missed by CT. The three patients with initial inguinal/iliacal LNM died during the observation period (overall survival: 10 (6–18) months). The three patients without inguinal/iliacal LNM involvement are currently alive, one patient showing a slowly progressive disease since 5 years, and two patients are tumor-free since 8.5 and 1.5 years (the patients had initial perirectal LNM).

**Conclusions:**

In ARMM, PET/CT is superior to CT in detection of LNM and distant metastasis. APR is possibly a curative approach if the PET/CT shows exclusively perirectal LNM despite locally advanced tumor growth.

## Background

Anorectal malignant melanomas (ARMM) are extremely rare and aggressive tumors representing 0.05 % of all malignant colorectal diseases and 0.5 to 4.6 % of all anorectal malignancies [1–4]. Despite an improvement of laboratory tests, as well as diagnostic imaging modalities and the development of multimodal therapy concepts in oncology, the prognosis of ARMM remains extremely poor. The median overall survival after diagnosis is specified to be between 8 and 19 months [5, 6]. Male gender, perineural invasion, infiltration depth of the rectal wall, lymph node metastasis (LNM), and distant metastasis are associated with a poor overall survival in ARMM [7–13]. LNM are detectable in more than 40 % of cases when an infiltration of the submucosa has occurred [9]. And, LNM is reported to be associated with a near to 0–5-year disease-specific survival [8]. This can be attributed to the fact that lymphatic spread in ARMM results in distant metastasis to the lung and liver in up to 90 % of cases [8, 12]. Until present, no patient has been reported to have survived more than 5 years in case of distant metastasis [14]. So although it is published that LNM in ARMM are associated with a poor overall survival, little is known about lymphatic spread to the inguinal/iliac lymph nodes or to the perirectal lymph nodes or to both regions, and if surgery should be adapted. The few existing data are inconsistent [15–17]. However, more than 10 different lymphatic pathways were described in the anal area [18], and a simultaneous presence of perirectal and inguinal LNM seems to be rare [19]. 18F-FDG-PET/CT scan is established in staging and follow-up examination of cutaneous melanomas [20]. Data about 18F-FDG-PET/CT in ARMM are sparse [21–26]. In general, the radical abdominoperineal resection (APR) [14] and the dissection of inguinal lymph nodes provide no survival benefit in ARMM [2]. The few long-term survivors described, however, often underwent APR [3, 9, 27, 28].

The objective of this study was to determine the pattern of inguinal/iliacal and perirectal lymph node metastasis by 18F-FDG-PET/CT or contrast-enhanced computed tomography (CT) in patients undergoing abdominoperineal resection and its impact on the oncologic outcome.

## Methods

### Patient group

A retrospective analysis of six consecutive patients who underwent abdominoperineal resection due to primary ARMM at the university hospital of Tuebingen between 2007 and 2014 was performed. The individual patients’ history, the pretreatment staging findings, and the radiologic findings are systematically listed in Tables 2 and 3. Demographic, diagnostic, surgery-related, and histopathologic parameters were examined using the clinic’s documentation system. The patients were investigated by contrast-enhanced whole-body CT and additionally by 18F-FDG-PET/CT (five patients) before surgery. The ARMM were categorized using a four-stage classification taking into account the depth of tumor infiltration (stage I and II), regional or lymphatic spread (stage III), and distant metastasis (stage IV) (Table 1 [14]). All patients were followed-up closely after surgery for up to 8.5 years.

**Table 1:** Staging classification of ARMM

Stage	Tumor spread
I	Local tumor spread without infiltration of the muscular layer
II	Local tumor spread with infiltration of the muscular layer
III	Regional tumor spread and/or positive lymph node metastasis
IV	Disseminated tumor spread

### Imaging

Patient preparation: after fasting for 12 h to ensure optimal glucose uptake during the examination, 350 MBq of 18F-FDG was injected intravenously. Patients were asked to rest during the uptake time to keep muscular glucose uptake on a low level. To facilitate the evaluation of the intestinal wall, 1000 ml 2.5 % mannitol solution was administered orally for intestinal distension. Before image acquisition, 40 mg butlyscopolaminiumbromid was injected intravenously to suppress intestinal motion, thus improving visualization of the intestine and closely surrounding structures and reducing misalignment of PET and CT images.

### Examination protocol

Due to the inclusion period of 7 years, patients were examined with a 16-slice whole-body PET/CT or with a 128-slice whole-body PET/CT (Hi-Rez Biograph 16/Biograph 128, Siemens Health Care, Knoxville TE, USA). Imaging techniques were comparable regarding image acquisition and diagnostic results. The technical parameters of the multidetector spiral CT and for PET have already been published by our research group [29]. Each examination consisted of a full diagnostic contrast-enhanced multidetector CT image acquired in the portovenous phase after injection of 80–120 ml iopromide (370 mg iodine/ml, flow rate 3 ml/s, Ultravist 370®, Bayer, Leverkusen, Germany), adjusted according to the patients’ body weight followed by a chaser of 40 ml saline and a high-resolution three-dimensional LSO PET covering 6–7 beds, depending on the patients’ size. The attenuation correction of PET data was performed using the contrast-enhanced CT images. PET image acquisition was started 1 h after 18F-FDG injection. CT images were reconstructed in transversal and coronal slice orientation with 5-mm slice thickness/5 mm increment in the transversal plane and 3-mm slice thickness/2 mm increment in the coronal plane, respectively.

### Image analysis

Image fusion of PET and CT data and evaluation of the separate CT and PET as well as fused PET/CT images were performed on a dedicated PET/CT workstation (TrueD, Siemens Health Care, Erlangen, Germany). Two experienced readers (specialists in radiology and nuclear medicine) evaluated CT and PET images separately, as well as the fused PET/CT images. This was done in a consensus mode for the presence of regional and distant metastases, as well as the extent of the primary tumor at the initial examination. For study purposes, a secondary analysis of CT and PET as well as merged PET/CT images was performed by a radiologist with 12 years of experience in oncologic imaging. To compare the diagnostic yield of CT and PET/CT, contrast-enhanced CT images of the PET/CT examination were assessed prior to reading the merged PET/CT image series to prevent a bias arising from the knowledge of the PET findings. Using CT data from the PET/CT examination ensures comparable disease stage, because acquisition took place at the same time.

## Results

The median age of the total cohort was 66.5 (range 54–73) years. Women were more often affected than men. All ARMM were localized to the dentate line, and tumor diameter varied between 0.4 and 11 cm at the time of diagnosis. In three patients, ARMM were diagnosed by biopsy and in the other three patients after performing local excision. In two patients the ARMM were amelanotic. KIT, BRAF, or NRAS mutations were not detectable in any patient. Serum levels of S100 protein was just elevated in patients having synchronous distant metastasis. Table 2 displays demographic data, tumor characteristics including histopathology, immunostaining, and mutation analysis as well as the therapy performed for each patient separately.

**Table 2:** Patient and therapy parameter, tumor characteristics, and follow-up data

	Patient 1	Patient 2	Patient 3	Patient 4	Patient 5	Patient 6
Age (years)	66	63	54	67	73	68
Gender	Male	Male	Female	Female	Female	Female
Diagnosis before abdominoperineal resection						
S100 protein in serum (normal range < 0.1 μg/l)	0.079	0.301	0.049	0.199	0.039	0.050
Preoperative biopsy	–	Yes	–	Yes	–	Yes
Previous local excision	Yes	–	Yes	–	Yes	
Therapy						
Type of abdominoperineal resection (APR)	APR	ELAPR	ELAPR	ELAPR	lap. ELAPR	lap. ELAPR
Intention for abdominoperineal resection	Curative	Palliative	Curative	Palliative	Palliative	Curative
Time interval between diagnosis and APR (months)	3	1	3	2	13	2
Adjuvant/additive therapy	–	DTIC	1. Ipilimumab	DTIC	1. Radiation	Ipilimumab (1 cycle)
			2. Nivolumab		2. DTIC	
					3. Ipilimumab	
Tumor characteristics, histopathology/immunostaining						
Tumor stage	I	IV	II	IV	III	III
Tumor localization referring to the dentate line	Above DL	Overlapping zones	On and above DL (multifocal)	DL	DL	Above DL
Tumor diameter (cm)	0.9	11	0.5 + 0.4 + 0.4	10.5	<1	10,9
Depth of infiltration (mm)	<3	15	4	All layers	3	All layers
Negative resection margin (R0)	Yes	Yes (<1 mm)	Yes	Yes	Yes (marginal)	Yes
Amelanotic melanoma	Yes	No	No	Yes	No	No
S100 protein	–	–	–	Positive	–	Positive
Melan-A	–	Positive	–	Positive	–	Positive
Mutation analysis						
KIT	–	Wild–type	Wild–type	Wild–type	Wild–type	Wild–type
BRAF	–	–	Wild–type	Wild–type	Wild–type	Wild–type
NRAS	–	–	Wild–type	Wild–type	–	Wild–type
Follow up (months)	102	6	60	10	18	18
Local recurrence	No	Yes	No	No	Yes	No
Time interval to abdominoperineal resection (months)	–	1	–	–	3	–
Metachronous lymph node metastasis	No	ns	Yes	Yes	Yes	No
Mediastinum	–	–	Yes	Yes	–	–
Perirectal	–	–	–	–	yes	–
Time interval to abdominoperineal resection (months)	–	–	42	3	5	–
Metachronous distant metastasis	No	ns	Yes	Yes	Yes	No
Lung/pleura	–	ns	Yes	–	Yes	No
Liver	–	–	No	Yes	Yes	No
Peritoneum	–	ns	No	No	Yes	No
Bone	–	ns	No	No	Yes	No
Soft tissue	–	ns	No	No	Yes	No
Brain	–	ns	No	No	Yes	No
Time interval to abdominoperineal resection (months)	–	–	21	3	12	–
Health status at follow-up time interval to abdominoperineal resection (months)	Disease-free	Dead 6	Slowly progressive disease	Dead 10	Dead 18	Disease-free

Before surgery, elevated serum levels of S-100 were measured only in patients with distant metastasis (stage IV). In all patients, the tumor origin was on or slightly above the dentate line. In patient 2, the tumor invades the anal canal. And, in patient 3, the ARMM was found at several localizations around the dentate line. During the first surgery, clear resection margins (R0) were achieved in all six patients. In patients 2 and 5, the resection margins were just barely R0. In patient 5, primarily, a local excision was performed. While in this patient multiple local recurrences were removed by local procedures over a time period of several months, also an LNM in the mesorectum was detected in the course. Consequently, laparoscopic extralevator abdominoperineal resection was carried out for local control. However, at this time, the tumor mass was not removable in total (R1). Patient 3 shows an atypical course of ARMM with a slowly progressive disease for more than 5 years

*APR* abdominoperineal resection, *ELAPR* extralevator abdominoperineal resection, *lap. ELAPR* laparoscopic extralevator abdominoperineal resection, *DTIC* dacarbacin, *DL* dentate line, *ns* not specified

**Fig. 1:**
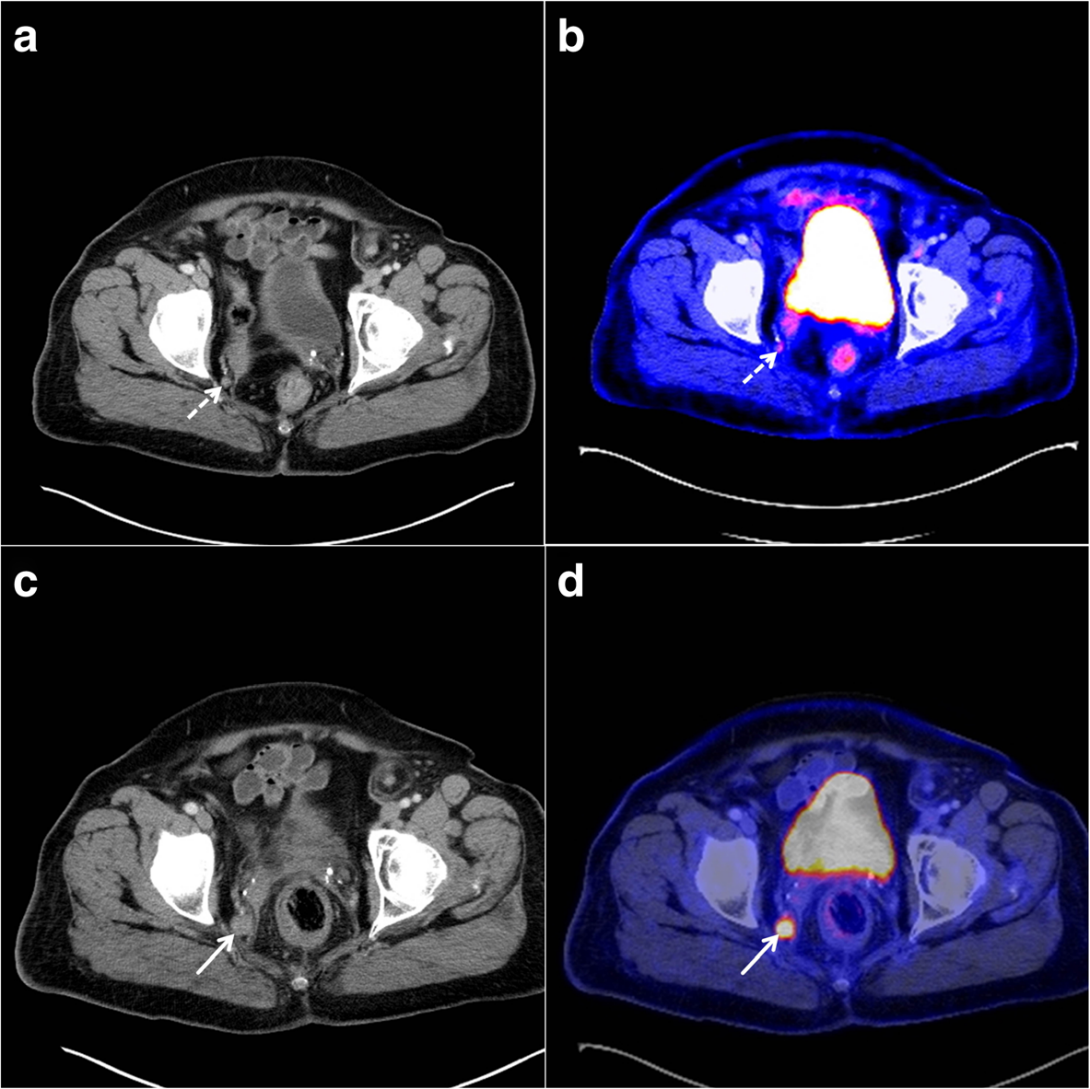
CT and PET/CT for pelvic imaging in anorectal malignant melanoma. A seventy-three-year-old female patient (*patient 5*) with a small iliacal lymph node metastasis on the right side (*dashed arrow*) and a relapse from anal melanoma. Contrast-enhanced CT reveals a small lymph node beside the right internal iliac artery of round configuration with uncertain dignity (**a**). PET/CT, however, indicates increased FDG uptake suspicious for metastasis (**b**). The follow up examination 9 months later provides clear evidence of progressive lymphatic metastasis on the right iliac side (*arrow*) on CT (**c**) and PET/CT (**d**) images. The lymph node is enlarged with a destroyed anatomical structure and increased glucose uptake. PET/CT enabled detection of lymph node metastasis in an early state

**Fig. 2:**
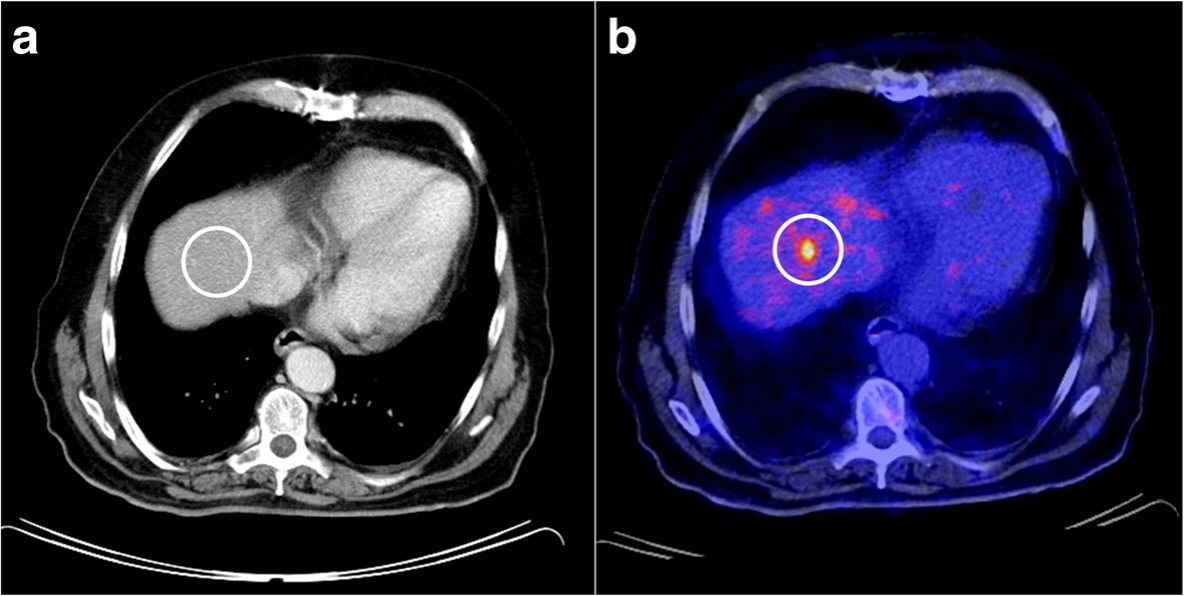
CT and PET/CT for hepatic imaging in anorectal malignant melanoma. A sixty-three-year-old male patient (*patient 2*) with a large anorectal melanoma, inguinal lymphatic metastases on both sides, and distant metastases. PET/CT depicts two hepatic metastases in segment six (**b**) and eight (*not in the picture*) without any corresponding findings on contrast-enhanced CT (**a**). Staging based on CT underestimated tumor stage by missing the distant metastatic spread completely in this patient

As tabulated in Table 3, four out of six patients had preoperative LNM involvement with two of them presenting with distant metastases additionally. In two patients, combined inguinal and perirectal LNM were determined. One patient had perirectal LNM exclusively and in another patient a solitary iliac LNM could be shown. During preoperative staging, inguinal/iliacal LNM in two patients as well as liver metastasis in one patient were seen in PET/CT and missed by CT. Also, when regarding metachronous metastases in the follow-up examination after surgery, in one patient, focally increased glucose uptake in the liver without correlating findings in CT were registered. On the other hand, in one patient, lymphonodular involvement was overestimated by CT which might arise from reactive lymph node enlargement without pathologic metabolic activity characteristic for melanoma metastases. Figure 1 shows examinations performed by CT and PET/CT where an iliac LNM could be diagnosed by PET/CT, but was not considered as suspect for LNM on CT imaging, and in Fig. 2, hepatic metastases were evident in the PET/CT examination, but not in the CT scan.

**Table 3:** Preoperative radiological staging and histopathological verification of lymph node metastasis in the mesorectum

	Patient 1			Patient 2			Patient 3			Patient 4			Patient 5			Patient 6		
	Occurrence	Detected in		Occurrence	Detected in		Occurrence	Detected in		Occurrence	Detected in		Occurrence	Detected in		Occurrence	Detected in	
		CT	PET/CT		CT	PET/CT		CT	PET/CT		CT	PET/CT		CT	PET/CT		CT	PET/CT
Synchronous LNM	No			Yes			No			Yes			Yes			Yes		
Perirectal	–			Yes	**X**	**X**	–			Yes	**X**	**X**	–			Yes	**X**	**X**
Inguinal right	–			Yes	**Ø**	**X**	–			–			–			–		
Iliacal right	–			–			–			–			Yes	**Ø**	**X**	–		
Inguinal left	–			Yes	**X**	**X**	–			Yes	**X**	**X**	–			–		
Iliacal left	–			–			–			–			–			–		
LNM in histopathology	No			Yes			No			Yes			Yes			Yes		
Perirectal (LNM/resLN)	0/12			13/33			0/14			1/3			(3/21)^a^			1/5		
Synchronous distant metastasis	No			Yes			No			Yes			No			No		
Lung/pleura	–			–			–			Yes	**X**	**X**	–			–		
Liver	–			Yes	**Ø**	**X**	–			–			–			–		

Detection of perirectal, inguinal, and iliacal lymph node metastases, as well as lung and liver metastases as depicted by contrast-enhanced CT and by 18F-FDG-PET/CT. Additionally, histopathologically verified lymph node metastases in relation to total count of examined lymph nodes are listed

*CT* computed tomography, *PET/CT* 18F-FDG PET/CT, *LNM* lymph node metastasis, *resLN* total count of resected lymph nodes

^a^Metachronous LNM, (X) metastatic lesions were detected, (Ø) metastatic lesions were not detected

Abdominoperineal resection was performed in five patients within 1 to 3 months after diagnosis and in one patient with a 13-month delay. In this patient, previous local excision was performed due to the initial assessment as a palliative situation in case of iliacal LNM involvement. Three patients underwent APR in palliative intention. Five patients received an adjuvant or additive therapy. In one patient, hepatic toxicity was detected early, so ipilimumab was canceled after one cycle. Only one patient underwent adjuvant radiotherapy (inguinal and pelvic).

Three patients died during the observation period (median survival: 10 (range 6–18) months). All three patients had synchronous inguinal or iliacal LNM. The other three patients did not have synchronous inguinal or iliacal LNM; one of them had perirectal LNM exclusively. Two patients are tumor-free since 8.5 and 1.5 years, including the patient who had synchronous perirectal LNM. The other patient shows a slowly progressive disease since 5 years. Nineteen months following extralevator abdominoperineal resection (ELAPR), a solitary pulmonary metastasis was diagnosed and resected. However, shortly afterwards, again, pulmonary metastases and mediastinal LNM were detected.

## Discussion

Despite a rapid development of diagnostic tools and advance in multimodal cancer therapy, the overall survival of ARMM has not substantially improved in the past decades. The lack of diagnostic and treatment standards is based on the rarity of this condition. An extended review of the literature, published in 2013 by our working group summarized the reported cases of ARMM to a total of 2650 [14]. In accordance to our patient population, the literature shows a high rate of LNM and distant metastases at initial diagnosis of ARMM and frequent metachronous metastases after a few months, which is associated with the poor overall survival [14]. In general, the overall survival cannot be improved by a radical surgical approach [14, 19, 30–32]. However, in the presented collective as well as in published studies, few long-term survivors seem to benefit from an abdominoperineal resection [3, 9, 27]. As far as no curative drug therapy is available, the identification of patients who could benefit from radical surgery (APR/ELAPR) is of paramount importance. For this, a diagnostic and treatment algorithm has been proposed [14]. Besides the individual tumor biology and perineural invasion, the lymphatic spread seems to have a key role affecting the poor overall survival [14, 33]. Systematic studies on the clinical relevance of LNM in ARMM are missing. And, the few existing data which try to structure the issue are quite inhomogeneous [19]. Analogous to the radical APR with total mesorectal excision, the groin lymph node dissection seems to have no influence on overall survival as well [2]. The reason for this might be found in the complex lymphatic drainage system of the anal region [27]. Up to now, more than 10 different lymphatic paths were found in the anal area [18]. Using a double-tracer technique (radiotracer and dye), Iddings et al. showed that all patients with ARMM drained to the inguinal node basin and even often to both sides [17]. This contrasts with historical studies on cadavers in which submucosal injections of quicksilver at the level of the dentate line shows a drainage to perirectal lymph nodes in 90–93 % [16]. Perez et al. published a rare simultaneous presence of perirectal and inguinal LNM [19]. Because of limitation of APR with total mesorectal excision on resection of perirectal LNM, only patients without LNM or with an exclusively perirectal lymphatic spread could benefit from radical surgery in curative intent. Information about non-invasive examination procedures such as the PET/CT in preoperative staging of ARMM, especially in detecting of lymphatic metastasis, is rare. Up to now, only a few case reports [21–25] and a case series of five patients [26] have been published. Several studies gave evidence that 18F-FDG-PET/CT provides superior diagnostic accuracy for the detection of metastases in malignant melanoma [20, 34–38] and in detecting recurrent colorectal carcinoma [39] compared to contrast-enhanced CT. The observations in our patient population also suggest that 18F-FDG-PET/CT is superior to the contrast-enhanced CT for detection of LNM and distant metastasis in some cases of ARMM. Altogether, in the preoperative staging, combined with the findings done during the follow up investigations, PET/CT identified distant metastases in two patients in the liver as well as metastatic lymph node involvement in two patients, which were all missed by contrast-enhanced CT. All LNM and distant metastasis described in PET/CT could be verified by histopathology (perirectal LNM) or have been confirmed in the short-term clinical course. On the other hand, in one patient lymphonodular involvement was overestimated by CT which might arise from reactive lymph node enlargement without pathologic metabolic activity characteristic for melanoma metastases. Although no statistical conclusions could be derived from these results due to the small number of patients, this supports the assumption that PET/CT contributes to improved diagnostic certainty in ARMM. Our observation confirms the descriptions of the only recently published collective, in which five patients with ARMM were examined preoperatively by PET/CT and by contrast-enhanced CT [26].

With regard to the primary tumor, PET/CT is of special interest. Tumor manifestations of the enteric wall tend to evade detection by morphologic assessment such as contrast-enhanced CT as lesions might be masked by stool or peristaltic muscle contraction or no circumscript tumor could be identified due to infiltrative growth. Especially after prior surgery, differentiation between scar tissue and new or recurring tumor is challenging. These patients will benefit from PET/CT to estimate tumor extent when considering surgical resection, especially in entities with high glucose consumption which usually is the case in malignant melanoma [29].

In our patient population, APR was performed in palliative intention in three patients. In each patient, inguinal or iliac LNM and distant metastases were evident. None of the three patients survived longer than 18 months after surgery. In contrast, during the observation period, three other patients survived, two of them without tumor recurrence. Interestingly, all three patients did not have any initial inguinal or iliac LNM, however, one patient had perirectal LNM exclusively. Already published data and the results in our patient population suggest that patients with initial inguinal/iliac LNM have no curative surgical option. Patients with perirectal LNM exclusively could benefit from curative APR even in the long term. Thus, the individual lymphatic anatomy of the anorectal region could be a decisive prognostic factor in ARMM and should be taken into account for patient selection for APR until no other valid data are available. Therefore, the 18F-FDG-PET/CT appears to be a useful tool for decision-making. In the three patients, an extralevator abdominoperineal resection was performed with palliative intention. The extent of resection was chosen analogous to the resection standards for very low rectal cancer in our university hospital. Due to the potentially higher morbidity of the extralevator abdominoperineal resection, the radicalness of surgery should be reduced to local tumor control in a palliative situation.

Due to the rarity of ARMM, no randomized controlled trials can be expected in the next few decades, particularly not answering all relevant questions concerning diagnosis and therapy of ARMM. Therefore, all patients with ARMM should be treated in specialized centers for recording data prospectively and to publish further case series. For a comparability of the data, we suggest a standardized recording, which is proposed by our working group [14].

## Conclusions

18F-FDG-PET/CT is superior to contrast-enhanced CT in staging of ARMM. LNM as well as distant metastasis, which might be missed by CT, was detected by PET/CT. Despite sparse data and a significantly higher cost burden compared to conventional diagnostics, PET/CT should be an obligatory part of the preoperative staging in ARMM. Patients, in which PET/CT shows perirectal LNM solely, could potentially benefit from curative abdominoperineal resection despite locally advanced tumor growth. Also, for staging in ARMM, there will be no randomized controlled data available in the foreseeable future. So, further extended case series should be published based on standardized collected data on PET/CT in ARMM.

## Abbreviations

APR, abdominoperineal Resection; CT, computed tomography; DL, dentate line; DTIC, dacarbacin; ELAPR, extralevator abdominoperineal resection; LNM, lymph node metastasis; 18F-FDG-PET/CT, 18F-fluordesoxyglucose positron emission tomography/computed tomography
